# A Challenging Case of Mammary Analogue Secretory Carcinoma: Case Study with Ultrastructural and Cytogenetic Correlation

**DOI:** 10.1155/2019/7468691

**Published:** 2019-12-17

**Authors:** Joseph Mathew, Michael Carvalho, Katherine Chorneyko, Samih Salama

**Affiliations:** ^1^Department of Pathology and Molecular Medicine, McMaster University/St. Joseph's Hospital, Hamilton Regional Laboratory Medicine Program, Hamilton, Ontario, Canada; ^2^Brant Community Healthcare System, Brantford General Hospital, Brantford, Ontario, Canada

## Abstract

Mammary analogue secretory carcinoma (MASC) is a rare salivary gland tumor analogous to secretory carcinoma of the breast. The diagnosis of MASC can be challenging due to substantial morphologic and immunohistochemical similarities with other salivary gland tumors. The differential diagnosis of MASC is broad and includes intraductal carcinoma, acinic cell carcinoma, and adenocarcinoma, not otherwise specified. Although molecular testing for ETV6 gene rearrangement is characteristic of MASC and has not been shown in any other salivary gland tumor, a particular challenge arises when such testing is unavailable, or when molecular testing for ETV6 gene rearrangement is negative in a suspected case of MASC. Our study presents the diagnostic workup of a challenging case of MASC with immunohistochemistry, electron microscopy, and cytogenetic studies performed to resolve the diagnosis.

## 1. Introduction

Mammary analogue secretory carcinoma (MASC), also known as secretory carcinoma of the salivary gland, is a rare tumor analogous to secretory carcinoma of the breast with identical morphology, immunohistochemical and molecular features including a characteristic t(12;15)(p15;q25) translocation resulting in the ETV6-NTRK3 fusion gene [1]. Unlike secretory carcinoma of the breast, which most often presents in pediatric patients, MASC typically presents in middle-aged adults and shows no sex predilection [2, 3]. Clinically, MASC most often manifests as a slow-growing painless mass in the parotid gland, although it has also been reported in the oral cavity and submandibular gland [2, 3]. MASC is generally considered to be a low-grade carcinoma with an overall favorable prognosis, although lymph node metastases are found in up to 25% of cases and rare cases of distant metastases have been reported [3]. Since the first publication describing MASC as a distinct entity in the salivary glands by Skálová et. al in 2010 [1], there has been a flurry of interest in this tumor including case series and literature reviews describing morphologic, immunohistochemical, and ultrastructural findings [2, 4–8]. However, the diagnosis remains challenging in certain cases; MASC can show considerable morphologic overlap with other salivary gland tumors including acinic cell carcinoma and intraductal carcinoma (formerly called low grade salivary duct carcinoma or cribriform cystadenocarcinoma) [9]. Although molecular testing for ETV6 gene rearrangement is characteristic of MASC and has not been shown in any other salivary gland tumor, a particular challenge arises when such testing is unavailable, or when molecular testing for ETV6 gene rearrangement is negative in a suspected case of MASC, which has been reported in some instances [1, 5]. Our study presents the diagnostic workup of a challenging case of MASC with immunohistochemistry, electron microscopy, and molecular findings.

## 2. Case Report

A 49-year-old female presented with a five-year history of a slowly growing, painless mass of the left parotid. CT scan of the head and neck demonstrated a partially cystic lesion of the superficial parotid, without involvement of nerves, vessels or lymph nodes. Fine needle aspiration cytology of the lesion showed sheets of epithelial cells with scant stroma in a cystic background suggestive of a salivary gland neoplasm of uncertain malignant potential.

### 2.1. Gross and Microscopic Pathology

The left parotid was completely excised, demonstrating a 1 cm well-circumscribed homogenous tan lesion with partially cystic areas on cut section. The remaining parotid gland was grossly unremarkable.

**Figure 1:**
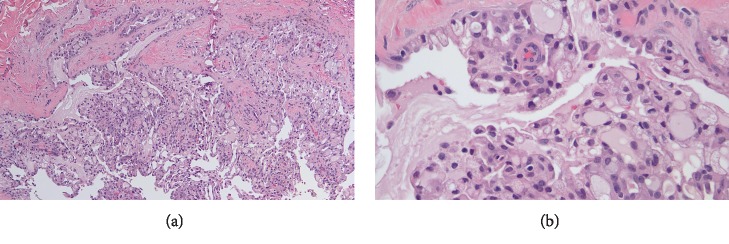
(a) H&E (100×). Characteristic cystic, papillary and cribriform architecture seen in mammary analogue secretory carcinoma (MASC). (b) H&E (400×) Characteristic cytologic features of MASC—cuboidal cells with abundant eosinophilic vacuolated cytoplasm, bland round to oval nuclei with inconspicuous nucleoli.

Routine H&E sections demonstrated a well-circumscribed predominantly intracystic epithelial salivary gland neoplasm with focal invasion. The tumor showed macrocystic and microcystic architecture as well as solid and papillary areas (Figure 1(a)). The lesional cells were cuboidal, with abundant eosinophilic vacuolated cytoplasm. The nuclei were round to oval, with homogenous chromatin and subtle nucleoli (Figure 1(b)). The resection margins were clear.

### 2.2. Immunohistochemistry

**Figure 2:**
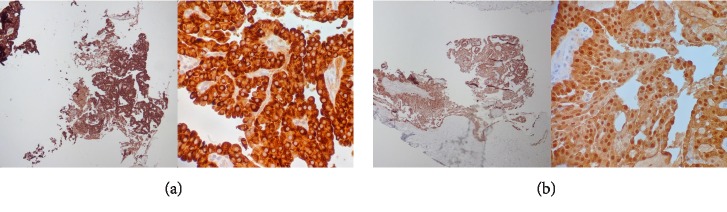
(a) Mammaglobin (Left—40x, Right—400x). (b) S100 (Left—40x, Right—400x).

The lesional cells were strongly and diffusely positive for mammaglobin (Figure 2(a)) and S100 (Figure 2(b)), as well as vimentin, CK7, CK19, BRST2, Cam5.2, and 34*β*E12. P63 showed focal nuclear staining in the lesional cells, while SMA highlighted scattered blood vessels confirming the absence of a surrounding myoepithelial layer. The tumor cells were negative for DOG1, CK5/6, EMA, CD117, and ER. Colloidal iron, Alcian blue, and PAS with and without diastase confirmed the presence of mucin.

### 2.3. Molecular Genetics

**Figure 3:**
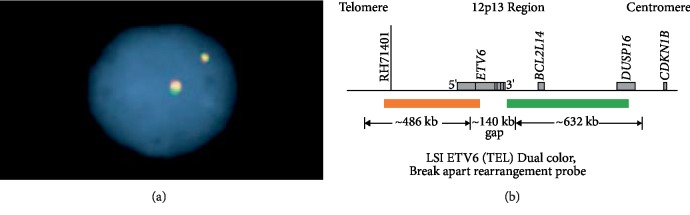
(a) Cell without ETV6 break-apart signal. (b) Probe map of the FISH kit used.

FISH failed to demonstrate a break apart of ETV6 (200 nuclei of lesional cells examined) (Figure 3).

### 2.4. Electron Microscopy

**Figure 4:**
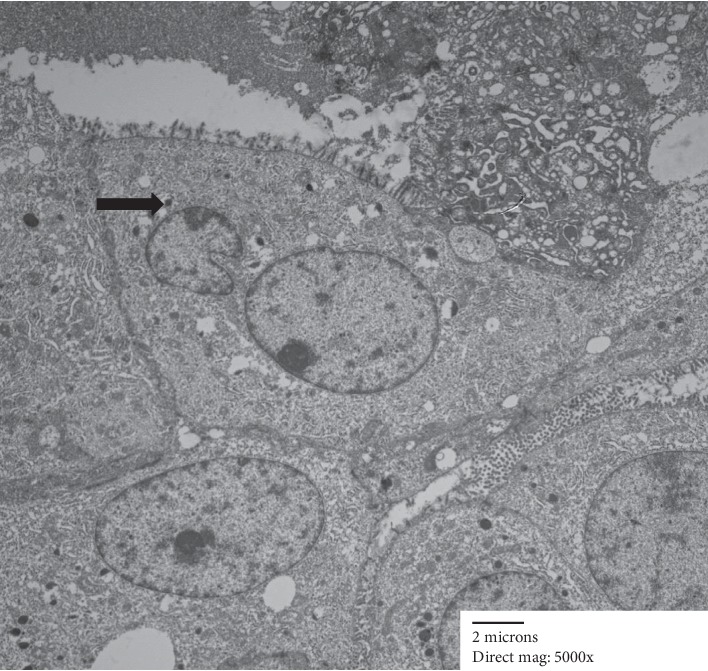
Lumina lined by short microvilli. Small dense granules (arrow) but no zymogen granules.

**Figure 5:**
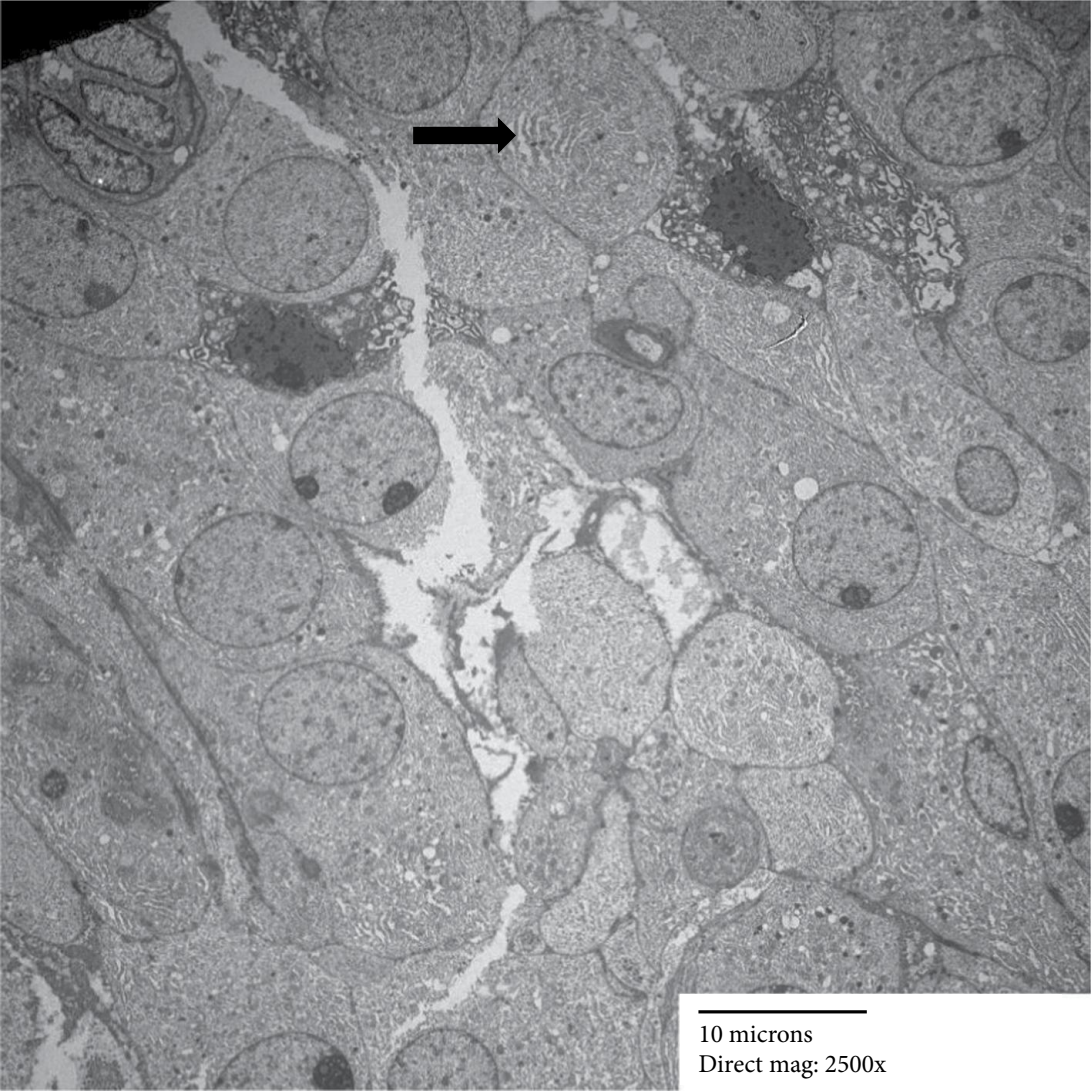
Nests of cells with slit-like luminal formation. Areas of prominent rough endoplasmic reticulum are seen (arrow).

**Figure 6:**
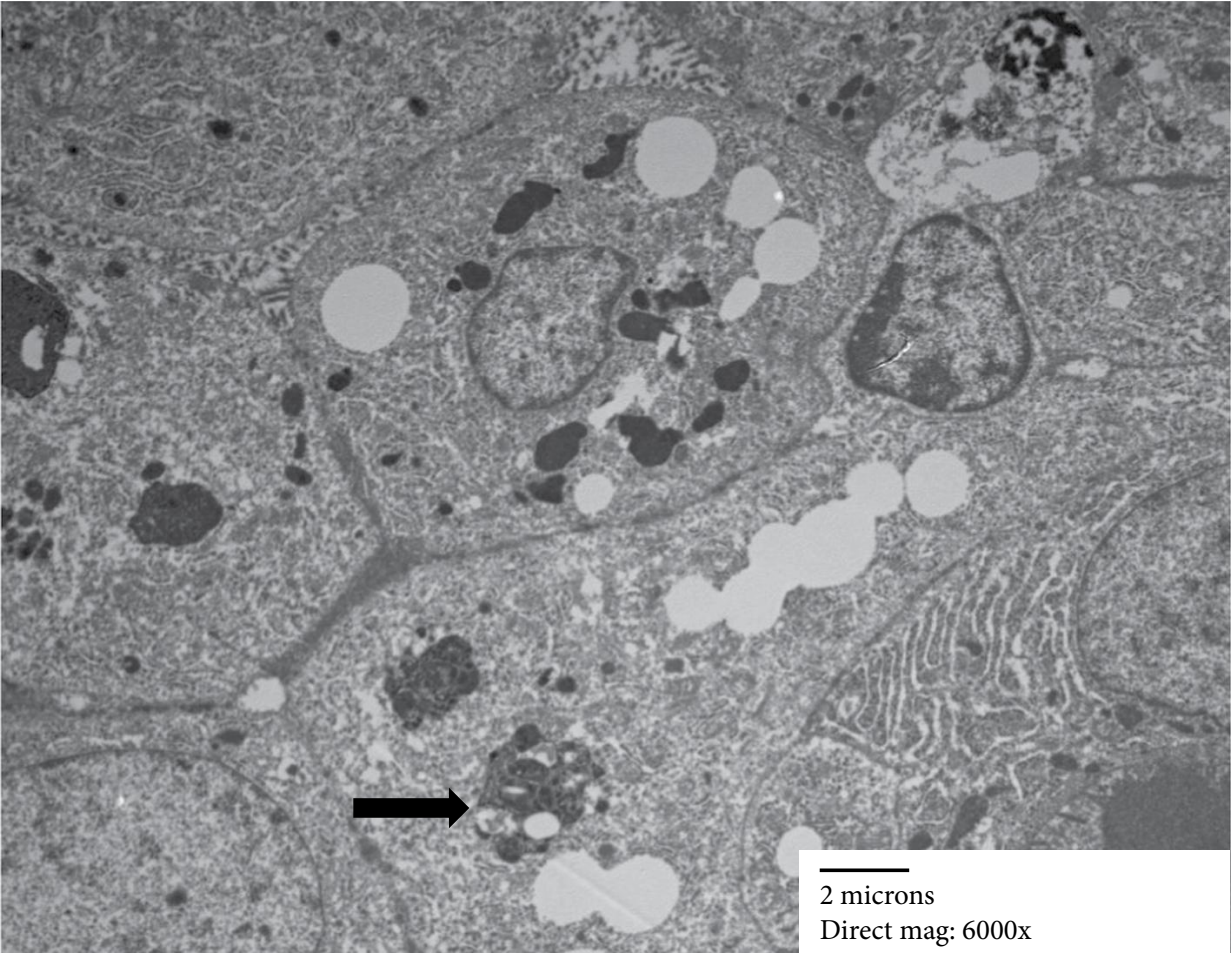
Nests of cells showing focal lipid, lysosomes (arrow) and stacks of rough endoplasmic reticulum.

Electron microscopy showed epithelial cells forming nests, with lumina lined by small microvilli (Figure 4). There was focally an abundance of rough endoplasmic reticulum (Figures 5 and 6). A few cells contained small intracytoplasmic, homogenous electron dense granules (Figure 4), however, diagnostic zymogen granules were not identified.

### 2.5. Clinical Followup

After 50 months of follow-up, the patient remains disease free, without clinical recurrence or metastasis.

## 3. Discussion

The diagnosis of mammary analogue secretory carcinoma (MASC) may be challenging due to substantial morphologic and immunohistochemical overlap with other salivary gland tumors. The differential diagnosis in our case included intraductal carcinoma (IDC, formerly called low grade salivary duct carcinoma or cribriform cystadenocarcinoma), acinic cell carcinoma, low-grade mucoepidermoid carcinoma and adenocarcinoma not otherwise specified (NOS). MASC can most often be differentiated from these tumors based on histology and immunohistochemistry as demonstrated in our case.

Intraductal carcinoma is a very rare salivary gland tumor with approximately 54 cases published to date [10]. Like MASC, it typically presents in the parotid gland [11], and has also been reported in the oral cavity, submandibular glands and minor salivary glands [10]. Overall, IDC has a more favorable prognosis compared with MASC, with no cases of regional or distant metastases of IDC reported to date [10]. IDC can show striking architectural resemblance to MASC with cystic, cribriform, papillary and solid architecture as well as eosinophilic secretions. There is a similar mirroring in the cytologic features—both having low-grade cuboidal cells with occasionally microvacuolated eosinophilic cytoplasm, round to oval nuclei, and inconspicuous nucleoli [11–13]. MASC and IDC show significant overlap in immunohistochemical profiles with the majority of both tumors showing diffuse positivity for S100 and mammaglobin [9, 12, 13]. The main distinction between IDC and MASC is the finding of predominantly intraductal growth in IDC which can be highlighted by myoepithelial cell markers such as P63, calponin or SMA showing a continuous rim of myoepithelial cells surrounding the tumor. Although IDC can show focal areas of invasion with loss of a corresponding myoepithelial layer, the majority of the tumor shows an intact myoepithelial layer consistent with intraductal growth [13]. In our case P63 showed very focal nuclear positivity in the lesional cells and SMA highlighted scattered blood vessels with no areas showing a continuous layer of intact myoepithelial cells, thus ruling out an intraductal growth pattern and supporting our diagnosis of MASC.

Like MASC, acinic cell carcinoma also presents most often in the parotid gland but is seen more commonly in females with a female-to-male ratio of 1.5 : 1 [14]. In comparison to MASC, ACC is more often associated with facial pain and facial paralysis. Histologically, acinic cell carcinoma can show solid, microcystic or papillary-cystic architecture similar to MASC, however, most cases of classical ACC can be easily distinguished from MASC by the presence of distinctive serous acinar cells with abundant basophilic granular cytoplasm containing PAS positive/diastase resistant zymogen granules [15]. Furthermore, these tumors show different immunohistochemical profiles—the vast majority of cases of ACC show diffuse strong cytoplasmic and membranous staining for DOG1 and negativity for S100 and mammaglobin. The greatest challenge in distinguishing ACC from MASC arises in cases of zymogen granule poor ACC, which have abundant eosinophilc vacuolated cytoplasm rather than granular basophilic cytoplasm and can also be positive for S100, although the negative staining for DOG1 and strong positive staining for mammaglobin seen in our case favors the diagnosis of MASC.

Low-grade mucoepidermoid carcinoma (MEC) may also be considered in the differential diagnosis of MASC. In low-grade MEC, well-differentiated mucinous cells may predominate over intermediate and squamoid cells, the two additional cell types common to MEC, and cystic architecture is also common in low-grade lesions, resulting in morphologic resemblance to MASC. Immunohistochemistry is helpful in distinguishing these two tumors, with p63 being positive in MEC and negative in MASC, as seen in our case.

Pleomorphic adenoma can occasionally have cystic architecture and characteristically shows bland cytologic features with cuboidal epithelial and myoepithelial cells with eosinophilic or clear cytoplasm, which can resemble the cytologic features of MASC. Pleomorphic adenoma is also positive for S100 in the myoepithelial component and can occasionally show positive staining for mammaglobin [4]. Unlike MASC, pleomorphic adenoma is associated with characteristic chondromyxoid stroma which was not seen in our case.

Adenocarcinoma NOS is a heterogeneous category of salivary gland tumors and ultimately a diagnosis of exclusion. Our case showed sufficient features to be categorized as MASC rather than adenocarcinoma NOS, including characteristic histology and immunophenotype.

Our study also adds to the limited number of reports describing the ultrastructural features of MASC. Only 3 studies on electron microscopic findings in MASC have been published to date [6, 7, 16]. As in the previous studies, our case showed the presence of small electron dense secretory granules which were not described in the single reported case of EM findings in intraductal carcinoma [17]. In addition, zygmogen granules characteristic of acinic cell carcinoma were not seen. Similar to previously reported EM findings in MASC, our case showed prominent rough endoplasmic reticulum as well as presence of lipid-laden vacuoles which have not been described in acinic cell carcinoma or IDC [16, 17]. As seen in this case, electron microscopy continues to have a role in further understanding the subcellular morphology of this tumor, and (if available) can help to exclude acinic cell carcinoma.

Although our case was negative for ETV6 gene rearrangement by FISH, we maintain that it is best classified as MASC based primarily on the classic histological and immunohistochemical findings. At least two cases of ETV6 translocation-negative MASC have been reported to date, including one case from the original case series describing MASC [1, 5]. There have been no reported differences in the histology or immunohistochemical profiles of ETV6 translocation-positive and translocation-negative cases. Furthermore, recent studies have supported the possibility of diagnosing MASC based on histology and immunohistochemistry alone, recognizing that molecular testing for ETV6 gene rearrangement may not be available in all centers, and that the accuracy of classification based on morphology and IHC has improved since the first description of MASC in 2010 [5]. Recently, studies have supported the use of pan-TRK immunohistochemistry as a sensitive and specific marker for NTRK fusions in MASC as well as other cancers [18–20]. Most importantly, our case highlights the importance of interpreting molecular results in the context of morphology and immunohistochemistry, in an era where confirmatory molecular testing is considered by some authors to be the gold standard in certain tumors.

The absence of ETV6 gene rearrangement by FISH in our case raises several considerations. Firstly, the possibility of a false negative result owing to a pre-analytical or analytical error cannot be entirely excluded, although FISH testing was performed following our clinical laboratory standard operating procedures and we carefully examined 200 tumor cells for ETV6 break apart signal reducing the possibility of a sampling error. Secondly, reports of rare cases of ETV6 negative MASC, including ours, raise the possibility that MASC may not harbor ETV6 rearrangement in all cases. Recent studies have shown an expanded molecular profile of MASC with ETV6 fusion partners other than NTRK identified, including RET [21], MET [22] and MAML3 [23]. Of note, our FISH assay utilized probes targeting both ends of the ETV6 gene and would have demonstrated a break apart signal regardless of the ETV6 fusion partner if an ETV6 rearrangement were present. Interestingly, a recent case reported by Black et al. showed an EGFR-SEPT14 fusion in addition to ETV6-RET fusion [24], the first reported case of MASC harboring a non-ETV6 gene fusion, supporting the possibility that oncogenic drivers other than ETV6 gene fusions may be involved in the pathogenesis of MASC. Further molecular analyses of MASC are required to definitively answer whether this tumor can develop in the absence of ETV6 gene rearrangement, with our study supporting this hypothesis.
